# Association between Obesity and Asthma in Preschool Mexican Children

**DOI:** 10.1100/tsw.2010.134

**Published:** 2010-07-07

**Authors:** Francisco Vázquez-Nava, Jaime Morales Romero, Arturo Córdova-Fernandez, Atenógenes H. Saldívar-González, Carlos F. Vázquez-Rodríguez, Maria del C. Barrientos-Gómez, Dolores Lin-Ochoa, Rodríguez Eliza M. Vázquez

**Affiliations:** ^1^ Department of Research in Clinical Epidemiology, No. 6 General Hospital Mexican Institute of Social Security, Tampico-Madero City, Mexico; ^2^ Public Health Institute, Veracruzana University, Veracruz, Mexico; ^3^ Department of Research, Faculty of Medicine, Autonomous University of Tamaulipas, Tampico, Mexico

**Keywords:** overweight, obesity, asthma

## Abstract

The elevated prevalence of obesity as well as of asthma in preschool children has prompted investigators to speculate that obesity in childhood might be a causal factor in the development of asthma. The results obtained to date are debatable. We investigated the association between obesity and asthma in 1,160 preschool Mexican children. Diagnosis of asthma was performed using the International Study of Asthma and Allergy in Childhood (ISAAC) questionnaire. The body mass index (BMI) in units of kg/m^2^ was determined, and children were categorized according to age- and gender-specific criteria, such as normal weight (5th-85^th^ percentile), overweight (ࣙ85^th^ and <95^th^ percentile), and obesity (ࣙ95^th^ percentile). Power test for logistic regression model was calculated. We found no association between overweight (adjusted OR = 1.02; 95% CI = 0.66–1.58), obesity (adjusted OR = 0.94; 95% CI = 0.68–1.30), and wheezing during the last year as determined by logistic regression model adjusted. We did not find an association between overweight, obesity, and asthma-associated hospitalizations. Further longitudinal studies are required to provide a better understanding of the relationship between obesity and asthma in preschool children.

## INTRODUCTION

Asthma and obesity constitute important health problems worldwide and have been related to a great number of negative health outcomes, particularly in children aged <5 years[1,2]. The estimated prevalence of asthma in preschool children is found within the range of 12–22%[3,4], and of asthma symptoms 20%[4]. The deleterious effects of asthma on quality of life and productivity in children has been documented previously[5,6,7,8,9].

Similarly, childhood obesity constitutes the most frequent nutritional problem of our time[10,11,12]. The results of the National Health and Nutrition Examination Survey 1999–2000 (NHANES) show that the prevalence of overweight in children between 2 and 5 years of age is 13.9%[12]. In México, the prevalence of overweight in preschool children is 10.4%[13]. Childhood obesity has been associated with an increased risk of some chronic diseases, including type 2 diabetes mellitus, high blood pressure, orthopedic difficulties, metabolic syndrome, and risk of obesity in adulthood[2,14,15].

Based on the prevalence of both asthma and obesity in childhood that has increased in recent decades, a possible connection between these two conditions has been suspected. At present, information available on the association between obesity and asthma in school children, adolescents, and the adult population is abundant, but debatable. Some researchers have found an association[16,17], but others have not[18,19]. Few studies have been conducted to determine the relationship between obesity and asthma restrictive to preschool children[20,21].

One priority for limiting the elevated prevalence and incidence of asthma is to identify the extent to which certain factors have an influence on the development of childhood asthma. Infancy is a life stage in which it is possible to study better the determinants of chronic diseases, such as asthma. Consequently, the present investigative work has been developed to determine the association between obesity and asthma in a cohort of 1,160 children aged 4–5 years.

## METHODS

We conducted a cross-sectional study based on a cohort of 1,160 children, 4–5 years of age (mean age = 4.5 ± 0.5 years), who had been followed longitudinally from the age of 4 months. Detailed descriptions of the sampling strategy have been described elsewhere[22]. Briefly stated, the study took place in the area of Tampico-Madero-Altamira, Mexico, located 542 km northwest of Mexico City.

The children who participated in this study were part of a population included in another major multidisciplinary research project in which a series of specialists took part, including a pediatrician, an allergy specialist, a family physician, dentists, general physicians, and dental assistants. Data analysis of the children included in this study was carried out in 2005. The children attended day care centers as required by the Scheme of Ordinary Participation and Unique Communitarian Neighborhood Program, organized by the Mexican Government to care for the children of working mothers through the Mexican Institute of Social Security (IMSS), and the preschool education school (kindergarten) of the area. The IMSS is the largest public health care institution in Mexico that provides care, pensions, and social security. Physical examinations are performed each month by family physicians, nursing personnel, nutritionists, etc. as part of the health care activities of the program of the day care center, and included are weight and height measurements, vaccinations, and the recording of contagious and noncontagious diseases detected. The personnel working at the IMSS day care centers are trained on an ongoing basis by experts by means of courses, talks, and workshops on the correct care of children. The day care center possesses sufficient information to identify and locate previously registered children, even after their departure at 4 years of age.

A questionnaire was utilized to register information on gender, asthma, and parental smoking [see Annex 1]. The questionnaire included closed multiple-choice and binomial questions, and was validated by two pilot studies. In each of these studies, 20 individuals were interviewed during a 15-day period; agreement was 75%.

The questionnaire was answered by the children's parents or guardians at the study site and was administered by an allergist, three dentists, and another duly trained individual. Training of the people who applied the questionnaire was carried out through talks and workshops on correct information gathering.

Questions on asthma were taken from the International Study of Asthma and Allergy in Children (ISAAC) questionnaire. The ISAAC program was developed to afford greater validity to epidemiological studies on asthma, allergic rhinoconjuntivitis, and atopic eczema[23].

**ANNEX 1**
**Items from ISAAC and ENSANUT-2006 Questionnaires used for the Logistic Regression**

1. Has your child ever had wheezing or whistling in the chest at any time in the past?
2. Has your child ever had wheezing or whistling in the chest during the past 12 months?
3. Has your child ever had a diagnosis of asthma by a physician?
4. In the last 12 months, has your child had a dry cough at night, apart from cough associated with a cold or a chest infection?
5. In the last 12 months, has your child's sleep been disturbed due to wheezing?
6. Number of hospitalizations or emergency service visits due to asthma in the last 12 months.
7. How many times a week does your child engage in a vigorous physical activity long enough to breathe hard?
8. How many hours a day (24 h) does your child watch television?
9. In the past 12 months, how often did your child eat or drink the following: fruit juices, soft drinks, potato chips, or candies?
10. Which of the parents smokes at home?

Additionally, parents were questioned concerning any prescription medication being administered to the children. We sought medication administered for asthma and classified these as inhaled and oral steroids, bronchodilators, or other medication.

We calculated the total energy intake and percentage of energy from fat, registering the type and amount of foods ingested during the 24 h prior to the study. Questions documenting the children's food-consumption habits and lifestyles were taken from the questionnaires applied at the ENSANUT-2006[13].

We employed a platform scale to weigh the children; this scale was calibrated prior to each weight measurement. Weighing was carried out with the child dressed in a minimum amount of clothing, which permitted the child to stand erect and relaxed. Weight was considered to the nearest 100 g. Height was measured via a stadiometer. This measurement was conducted with the child barefoot, maintaining the head in a neutral position, with the neck, spinal column, and knees in physiological extension and the soles of both feet totally supported on a horizontal surface.

Body mass index (BMI = $kg/m^2$) was determined using the age- and gender-specific Centers for Disease Control and Prevention (CDC) definition[24]. Children were categorized as follows: normal weight: 5–85$^{th}$ percentile; overweight: ≥85$^{th}$ and <95$^{th}$ percentile; and obesity: ≥95$^{th}$ percentile. For analysis, these variables were coded as:

| | |
|---|---|
| 1 = Abnormal weight | 0 = Normal weight |
| 1 = Overweight | 0 = Normal weight |
| 1 = Obesity | 0 = Normal weight |

Children diagnosed with asthma, overweight, obesity, or any other pathology were referred for care of their disease(s) to the corresponding Medical Service Unit. Parental written, oral, and informed consent was requested and obtained.

The study was approved by the Ethics Committee of the No. 6 General Hospital of the IMSS and the Faculty of Medicine of the Autonomous University of Tamaulipas (UAT), Mexico.

## Statistical Analysis

Data were analyzed by the SPSS 10.0 statistical package software. We utilized dichotomous variables for indicating presence or absence of a characteristic. Single frequencies and central tendency measurements

(medians and standard deviations [SD]) were employed. A 2 × 2 contingency table ($\chi^2$, odds ratio [OR], and a 95% confidence interval [95% CI]) were utilized to ascertain asthma risks caused by the effect of obesity by comparing a group with asthma and another without it. Logistic regression models were performed to study the association between obesity and asthma**.** In evaluating the association, we adjusted for the following confounders: gender and smoking parents in the home. Any *p* value <0.05 was considered significant. Power test for logistic regression model was calculated using the method proposed by Hsieh et al.[25]. Power test was calculated for an OR of two, using the number of cases and the proportion of cases on each class according to each analysis.

## RESULTS

Asthma prevalence was 5.3 and 19.5% (n = 226) of children who experienced wheezing during the last 12 months. Age at initiation of asthma was 14.53 ± 12.23 months. Approximately 14.2% (n = 165) of the children were overweight and 32.1% (n = 372) suffered from obesity. A group of 623 children was identified as having normal weight. These children were used as the control group for the analysis in order to detect a difference between normal and abnormal weight. Table 1 presents the distribution by gender of the prevalence of asthma, wheezing ever in their life, wheezing during the last year, dry cough during the night, hospitalizations due to asthma, normal weight, overweight, and obesity. The prevalence of asthma was similar in boys (5.0%) and girls (5.5%). A higher percentage of wheezing during the last year (21.5 vs. 17.5%) was found in males than in females. Overweight (17.1 vs. 11.3%) and obesity (33.2 vs. 30.9%) prevalence was higher in females than in males.

**TABLE 1**
**Distribution by Gender**

| | Male | | Female | | Total Sample | |
|---|---|---|---|---|---|---|
| | n | % | n | % | n | % |
| Asthma diagnosis by a physician | 29 | 5.0 | 32 | 5.5 | 61 | 5.3 |
| Wheezing ever in their life | 122 | 21.0 | 99 | 17.1 | 221 | 19.1 |
| Wheezing during the last year | 125 | 21.5 | 101 | 17.5 | 226 | 19.5 |
| Dry cough at night | 43 | 7.4 | 33 | 5.7 | 76 | 6.6 |
| Sleep disturbance due to wheezing | 45 | 7.8 | 30 | 5.2 | 75 | 6.5 |
| Hospitalization due to asthma | 17 | 2.9 | 8 | 1.4 | 25 | 2.2 |
| Normal weight | 336 | 57.7 | 287 | 49.7 | 623 | 53.7 |
| Overweight | 66 | 11.3 | 99 | 17.1 | 165 | 14.2 |
| Obesity | 180 | 30.9 | 192 | 33.2 | 372 | 32.1 |

Table 2 displays the percentage of overweight and obesity in children that suffered from asthma, wheezing, dry cough during the night, and hospitalizations due to asthma during the last 12 months. There was a slightly greater prevalence of asthma among the children with overweight (7.3 vs. 4.7%) than those without it. The prevalence of obesity was similar among the children with asthma (5.4%) than those without it (4.7%).

In the multivariate logistic regression model (Table 3), overweight (adjusted OR = 1.58; 95% CI = 0.78–3.19) and obesity (adjusted OR = 1.16; 95% CI = 0.64–2.08) were not associated with asthma.

**TABLE 2**
**Prevalence of Overweight and Obesity**

| | Asthma Diagnosis | | Wheezing Ever | | Wheezing Last Year | | Dry Cough | | Hospitalization | |
|---|---|---|---|---|---|---|---|---|---|---|
| | n | % | n | % | n | % | n | % | n | % |
| Females | 29/553 | 5.0 | 122/460 | 21.0 | 125/457 | 21.5 | 43/539 | 7.4 | 17/565 | 2.9 |
| Males | 32/546 | 4.5 | 99/479 | 17.1 | 101/477 | 17.5 | 33/545 | 5.7 | 8/570 | 1.4 |
| Normal weight | 29/594 | 4.7 | 123/500 | 19.7 | 124/499 | 19.9 | 49/574 | 7.9 | 19/604 | 3.0 |
| Abnormal weight | 32/505 | 6.0 | 98/439 | 18.2 | 102/435 | 19.0 | 27/510 | 5.0 | 6/531 | 1.1 |
| Overweight | 12/153 | 7.3 | 36/129 | 21.8 | 32/133 | 19.4 | 13/152 | 7.9 | 2/163 | 1.2 |
| Obesity | 20/352 | 5.4 | 62/310 | 16.7 | 70/302 | 18.8 | 14/358 | 3.8 | 4/368 | 1.1 |

**TABLE 3**
**Association between Overweight, Obesity, and Asthma-Associated Concerns**

| | Asthma Diagnosis | Wheezing Ever | Wheezing Last Year | Dry Cough at Night | Hospitalization |
|---|---|---|---|---|---|
| | Adjusted OR (95% CI)[a] (Significance and Power Level)[b] | | | | |
| Females[c1] | 1.07 (0.64–1.81)** | 0.77 (0.57–2.03)** | 0.70 (0.57–1.03)** | 0.76 (0.47–1.22)** | 0.48 (0.20–1.14)** |
| Normal weight[c2] | 0.77 (0.46–1.30)** | 1.08 (0.80–1.45)** | 1.04 (0.77–1.39)** | 1.58 (0.97–2.58)** | 2.67 (1.06–6.76)* |
| | (0.3414;0.9015) | (0.6011;0.9999) | (0.7805;0.9999) | (0.0626;0.9825) | (0.0379;0.7640) |
| Overweight[c3] | 1.58 (0.78–3.19)** | 1.17 (0.77–1.79)** | 1.02 (0.66–1.58)** | 1.02 (0.54–1.95)** | 0.41 (0.09–1.80)** |
| | (0.1993;0.5855) | (0.4501;0.9797) | (0.9171;0.9800) | (0.9304;0.7936) | (0.2416;0.4173) |
| Obesity[c3] | 1.16 (0.64–2.08)** | 0.82 (0.58–1.15)** | 0.94 (0.68–1.30)** | 0.46 (0.25–0.85)* | 0.35 (0.11–1.04)** |
| | (0.6204;0.8347) | (0.2528;0.9995) | (0.6974;0.9995) | (0.0135;0.9580) | (0.0609;0.6716) |

[a] Adjusted for gender and smoking parents in the home.

[b] *p* value (***p* > 0.05; **p* < 0.05); power calculated to detect as significant (*p* = 0.05) an odds ratio of two. Number of cases and percentage of cases in each class used were shown in Table 2.

[c] Reference category: c1, males; c2, abnormal weight; c3, normal weight.

We found practically no differences in the prevalence of wheezing during the last year (Table 2) in children who suffered from overweight (19.4 vs. 19.9%) or obesity (18.8 vs. 19.9%) than those with normal weight. Overweight (adjusted OR = 1.02; 95% CI = 0.66–1.58) and obesity (adjusted OR = 0.94; 95% CI = 0.68–1.30) were not associated with wheezing during the last year as determined by logistic regression model adjusted (Table 3).

The only two significant models were those that relate normal weight with hospitalization and obesity with dry cough at night; neither of both are a risk (Table 3).

The sample size used gives high power to test OR (at $p = 0.05$ and OR = 2). Most of analyzed models showed a good power larger than 0.83 (Table 3).

## DISCUSSION

In this study employing a cohort of 1,160 preschool children aged 4–5 years, we provide evidence that overweight and obesity are not associated with asthma in preschool children. Previous studies are in agreement with our results. Kwon et al.[26] studied 253 children aged 2–5 years. The authors found that obesity is not associated with asthma in preschool children. However, other researchers, such as von Kries et al.[21] in their study of 9,317 children aged 5–6 years, found that overweight and obesity are associated with asthma. It is possible that the different results are related to the differences in the prevalence of asthma in the studied populations or to the sample size that we studied.

The mechanisms that determine the relationship between obesity and asthma in preschool children are unclear. The association between obesity and asthma in school children, adolescents, and the adult population has been attributed to hormonal factors, lifestyle, different eating habits, and airway size[16,20,27,28,29]. It is probable that some of these factors can be functioning in preschool children. Additional longitudinal research studies are required to prove this hypothesis.

According to recent reports, the prevalence of childhood asthma[4] and childhood obesity continues to increase[10,11]. In our study, the percentage of asthma, overweight, and obesity was similar to that reported in recent studies[3,4].

Previous investigations showed that overweight and obesity, are not associated with severity of asthma, determined according to the presence of asthma symptoms, such as wheezing-related sleep disturbances or dry cough at night during the last 12 months[16,21]. In our study, the percentage of children with asthma symptoms was lower than that reported by other authors[3,4]. Approximately 6.5% reported wheezing-associated sleep disturbances and 6.6% had dry cough at night during the 12 months prior to the study. The analyses support the hypothesis that neither overweight nor obesity are associated with dry cough at night during the last 12 months in children <5 years of age.

In this study, 2.2% of children were hospitalized due to asthma in the previous year. Children who were normal weight were hospitalized more as compared with children who were overweight (3.0 vs. 1.2%) and obese (3.0 vs. 1.1%). Based on our data, overweight and obesity are not associated with asthma-related hospitalization. Nonetheless, other investigators, such as Sin et al.[30], demonstrated that individuals of the masculine gender with severe asthma who require emergency services tend to be more obese. The mechanisms by which overweight or obesity are related to a greater prevalence of hospitalization in males is unclear. Some researchers have shown that males have shorter airways relative to lung size[31]. On the other hand, Sherman and colleagues[9], on studying early childhood predictors of asthma in a cohort of 770 children aged 5–9 years, found that males with asthma had a larger predicted FVC percentage, while females with asthma had a lower predicted $FEV_1$ percentage than their counterparts, suggesting, thus, that there is clear male/female evidence for the effect of asthma on lung function level.

This study possesses certain limitations that should be taken into consideration on generalizing its results as follows: (1) Information concerning asthma and asthma symptoms in the current study was derived from self-reports provided by the children's parents using the ISAAC questionnaire. However, several studies have shown that this instrument can be considered effective for measuring diagnosis of asthma[32]. (2) These results cannot be generalized to the open population because the children participating in the study attend day care centers and preschool education schools that have medical services and nutrition programs.

We conclude that overweight and obesity are not associated with asthma in preschool children. Further longitudinal studies are required to provide a better understanding of the relationship among overweight, obesity, and asthma in preschool children.
